# Characterisation of PVL-Positive *Staphylococcus argenteus* from the United Arab Emirates

**DOI:** 10.3390/antibiotics13050401

**Published:** 2024-04-27

**Authors:** Stefan Monecke, Sindy Burgold-Voigt, Sascha D. Braun, Celia Diezel, Elisabeth M. Liebler-Tenorio, Elke Müller, Rania Nassar, Martin Reinicke, Annett Reissig, Abiola Senok, Ralf Ehricht

**Affiliations:** 1 Leibniz Institute of Photonic Technology (IPHT), Leibniz Center for Photonics in Infection Research (LPI), 07745 Jena, Germanyannett.reissig@leibniz-ipht.de (A.R.); ralf.ehricht@leibniz-ipht.de (R.E.); 2 InfectoGnostics Research Campus, 07743 Jena, Germany; 3 Friedrich-Loeffler-Institut, Institute of Molecular Pathogenesis, 07745 Jena, Germany; 4 College of Medicine, Mohammed Bin Rashid University of Medicine and Health Sciences, Dubai P.O. Box 505055, United Arab Emiratesabiola.senok@mbru.ac.ae (A.S.); 5 School of Dentistry, Cardiff University, Cardiff CF14 4XY, UK; 6 Institute of Physical Chemistry, Friedrich-Schiller University, 07743 Jena, Germany

**Keywords:** *Staphylococcus argenteus*, *Staphylococcus aureus*, Panton–Valentine leukocidin, prophages, phage induction, microarray, nanopore sequencing, transmission electron microscopy

## Abstract

*Staphylococcus argenteus* is a recently described staphylococcal species that is related to *Staphylococcus aureus* but lacks the staphyloxanthin operon. It is able to acquire both resistance markers such as the SCC*mec* elements and mobile genetic elements carrying virulence-associated genes from *S. aureus*. This includes those encoding the Panton–Valentine leukocidin (PVL), which is associated mainly with severe and/or recurrent staphylococcal skin and soft tissue infections. Here, we describe the genome sequences of two PVL-positive, *mecA*-negative *S. argenteus* sequence type (ST) 2250 isolates from the United Arab Emirates in detail. The isolates were found in a dental clinic in the United Arab Emirates (UAE). Both were sequenced using Oxford Nanopore Technology (ONT). This demonstrated the presence of temperate bacteriophages in the staphylococcal genomes, including a PVL prophage. It was essentially identical to the published sequence of phiSa2wa_st78 (GenBank NC_055048), a PVL phage from an Australian *S. aureus* clonal complex (CC) 88 isolate. Besides the PVL prophage, one isolate carried another prophage and the second isolate carried two additional prophages, whereby the region between these two prophages was inverted. This “flipped” region comprised about 1,083,000 bp, or more than a third of the strain’s genome, and it included the PVL prophage. Prophages were induced by Mitomycin C treatment and subjected to transmission electron microscopy (TEM). This yielded, in accordance to the sequencing results, one or, respectively, two distinct populations of icosahedral phages. It also showed prolate phages which presumptively might be identified as the PVL phage. This observation highlights the significance bacteriophages have as agents of horizontal gene transfer as well as the need for monitoring emerging staphylococcal strains, especially in cosmopolitan settings such as the UAE.

## 1. Introduction

In 2006, methicillin-resistant *Staphylococcus aureus* (MRSA) outbreaks among remote Aboriginal communities of northern regions of Australia were described [1]. These strains, usually carrying SCC*mec* IV elements, were assigned to the multilocus sequence type ST75, and in West Australia (WA), they were dubbed “WA-MRSA-8”. An obviously similar strain, also from remote Australian regions, was ST883 (“WA-MRSA-47”). It soon became clear that these lineages differed from ordinary *S. aureus* lineages in having distinct alleles of crucial genes [2,3]. As primers for multilocus sequence typing which were matched poorly, the MLST scheme and its primer sequences were modified [2,4,5], resulting in a re-assignment, with ST75 eventually becoming ST1850 and ST883 becoming ST2198. As these strains also lacked the “golden” carotenoid pigment staphyloxanthin (which is so typical for *S. aureus* that it was even the reason for naming it *“aureus*” [6]), they were described as a novel species that consequently was named *S. argenteus*, i.e., the “silver-coloured” Staphylococcus [5].

In addition to the distribution in Australia, many reports of a presence of *S. argenteus* from other countries were published. It was found in New Zealand [7] and on the Fiji Islands [7,8]. *S. argenteus* is widely distributed in Asia, being observed in Singapore [9], Cambodia [4], Laos [10], Myanmar [11], Thailand [12,13], China [14], Taiwan [14,15], and Japan [16,17,18]. French patients with *S. argenteus* were shown to have epidemiological links to the Indian Ocean island of Mayotte [19]. *S. argenteus* was also found in Gabon [20], Trinidad and Tobago [21], French Guiana [22], and Brazil [23]. Sporadic observations, presumably associated with travel and migration, originate from the United Arab Emirates [24], several European countries, and Israel [19,25,26,27,28,29,30,31], as well as from the United States of America and Canada [32].

*S. argenteus* has also been identified in animals such as rabbits [12], dairy cattle [13,23], a dog [33], macaques (*Macaca fascicularis* [34]), and a gorilla (*Gorilla gorilla* [20]), but strains from Nigerian bats should be assigned to another species [35,36].

While *S. argenteus* can be asymptomatically carried in the nares, it can essentially cause the same infections as *S. aureus* [10,30,37,38,39], including skin and soft tissue infections [10,17], osteomyelitis [25], or endoprosthesis infections [27,29] and sepsis [14,18]. In vitro, it appears to be as cytotoxic as *S. aureus* [40]. *S. argenteus* has also been described as a cause of food poisoning [41].

Genetically, this species is closely related to *S. aureus*, but also to other species from the “coagulase-positive *S. aureus*-like” complex (which, in addition to *S. aureus* and *S. argenteus,* also includes *S. schweitzeri* [42] and *S. roterodami/singaporensis* [9,35,43]). All can be typed and distinguished using *S. aureus* MLST primers, and all carry alleles of the same core genome genes, arranged in the same order and orientation of their genomes. *S. argenteus* can be divided into MLST-defined clonal complexes, which, as in *S. aureus*, also differ in the presence of genomic islands [44]. Thus, ST1223, ST1850 (formerly ST75), ST2198 (formerly ST883), ST2250, ST2596/2793, ST2854, and ST4587 can be regarded as founder STs of homonymous *S. argenteus* clonal complexes (CC*_arg_*). Some mobile genetic elements previously known from *S. aureus* have also been identified in *S. argenteus*. This includes SCC*mec* IV and V elements that carry the methicillin/beta-lactam resistance gene *mecA*; the pathogenicity-island-borne toxic shock syndrome toxin *tst1* [45]; enterotoxins such as *seb* [46], *sed+sej+ser* [46], and *seh* (JABA32044V6S1; GenBank CCEE/SAMEA2007996); and phages harbouring the Panton–Valentine leukocidin, PVL [19,24,45].

PVL consists of two proteins (LukF-PV and LukS-PV) encoded by two co-expressed genes (*lukF*-PV and *lukS*-PV) localised on *Siphoviridae* prophages [47,48,49,50,51]. These lysogenic phages/prophages play a crucial role in horizontal gene transfer, transferring antimicrobial resistance and virulence determinants to other enabling bacteria to adapt to environmental challenges posed by host defences. PVL components form polymeric pores in leukocyte membranes, leading to cell death. PVL is usually associated with severe, chronic, or recurrent skin and soft tissue infections [52], but it also might cause life-threatening conditions such as necrotising pneumonia. Because of its phage-borne nature, it can be found in many different lineages of *S. aureus.* In general, PVL-positive *S. aureus* is common in Africa, the Indian Subcontinent, the Middle East, Australia, the Caribbean, and the US, but is rather rare in Northern and Western Europe. Apparently, there were pandemics of PVL-positive *S. aureus* strains in the 1930s and 1960s, while during the last 25 years, a multitude of methicillin-resistant *S. aureus* emerged that, in addition to *mecA*/SCC*mec,* also carried PVL prophages [46,53]. These became a major public health problem in some parts of the world, namely, the US, Australia, the Middle East, and the Indian Subcontinent.

Here, we describe the genome sequences of two PVL-positive but methicillin-susceptible *S. argenteus* isolates from the United Arab Emirates and characterise their prophages, including the ones carrying the PVL genes.

## 2. Results

### 2.1. Identification as PVL-Positive CC_arg_2250 by Microarray and Nanopore Sequencing

Two isolates, Dubai-25 and Dubai-30, were found during a previously published study on nasal colonization and environmental contamination in academic dental clinics in the UAE [24]. Both isolates were obtained from the same academic dental centre. Dubai-25 was obtained from a nasal swab of a student of Middle Eastern descent, while Dubai-30 was obtained from an environmental surface in the dental clinic. Samples were obtained on 20 November 2018. The swabs were processed in the laboratory by vortexing them individually in brain heart infusion broth (BHI) containing 6.5% NaCl for 2 min, followed by incubation of the broth at 35–37 °C for 24–48 h. Subculture was carried out from turbid BHI tubes onto mannitol-salt agar (MSA). Directly upon isolation, *S. aureus* was identified using conventional methods, i.e., VITEK2 (BioMerieux, Marcy L’Etoile, France) and MALDI-TOF (Bruker Daltonics, Bremen, Germany). Later, isolates were identified as *S. argenteus* via microarray analysis, which also allowed for the detection of PVL genes and an assignment to CC*_arg_*2250 [24].

With regard to microarray profiles, both isolates lacked the Staphyloxanthin operon genes. They did not yield hybridisation signals for *agrB/C/D* I to IV probes, but sequencing showed the presence of deviant alleles, as was previously observed for other *S. argenteus* (see [3] and GenBank CP023076.1 with *agrB*, CJ017_09870; *agrC*, CJ017_09880; and *agrD*, CJ017_09875), but they harboured *gapA, nuc1, eno,* and *fnbA*. Furthermore, they harboured *sasG*, while the *egc* enterotoxin gene cluster and the collagen adhesin gene *cna* were absent. This was consistent with an affiliation to CC*_arg_*2250, as represented by the strain XNO62, GenBank CP023076.1. Once the genome sequences (Supplemental File S1) were obtained, MLST typing was performed using the PubMLST database. Both isolates yielded the profile *arcC*-151, *aroE*-325, *glpF*-215, *gmk*-34, *pta*-175, *tpi*-180, *yqiL*-169, that is, ST2250.

Both isolates were positive for the PVL genes. They lacked exfoliative toxin genes, *tst*1, and the classical enterotoxin genes. Genome sequencing, however, revealed a presence of two putative enterotoxin genes (“*seli2=sel26*” and “*selu2=sel27*”) known from CC*_arg_*2250: CCEM01000001 (985,525…986,250) and (986,277…987,029); CP023076.1 (1,842,698…1,843,415) and (1,843,450…1,844,202); as well as *S. schweitzeri,* LR134304 (1,887,932…1,888,657) and (1,888,684…1,889,436).

Neither isolate harboured any antibiotic resistance genes, and they lacked SCC*mec* elements. However, genome sequencing showed a presence of a CRISP/*cas* element at the position normally inhabited by SCC elements, downstream of *orfX,* which confirmed the array-based observation of *cas1* (SAMSHR1132_00520) and which is in accordance with previously published *S. argenteus* genomes (locus tags SAMSHR1132_00490 to 00610 in the CC*_arg_*1850 sequence FR821777 and CJ017_00150 to 00205 in CC*_arg_*2250, CP023076).

### 2.2. Inversion of a Part of the Genome in One Isolate

**Figure 1:**
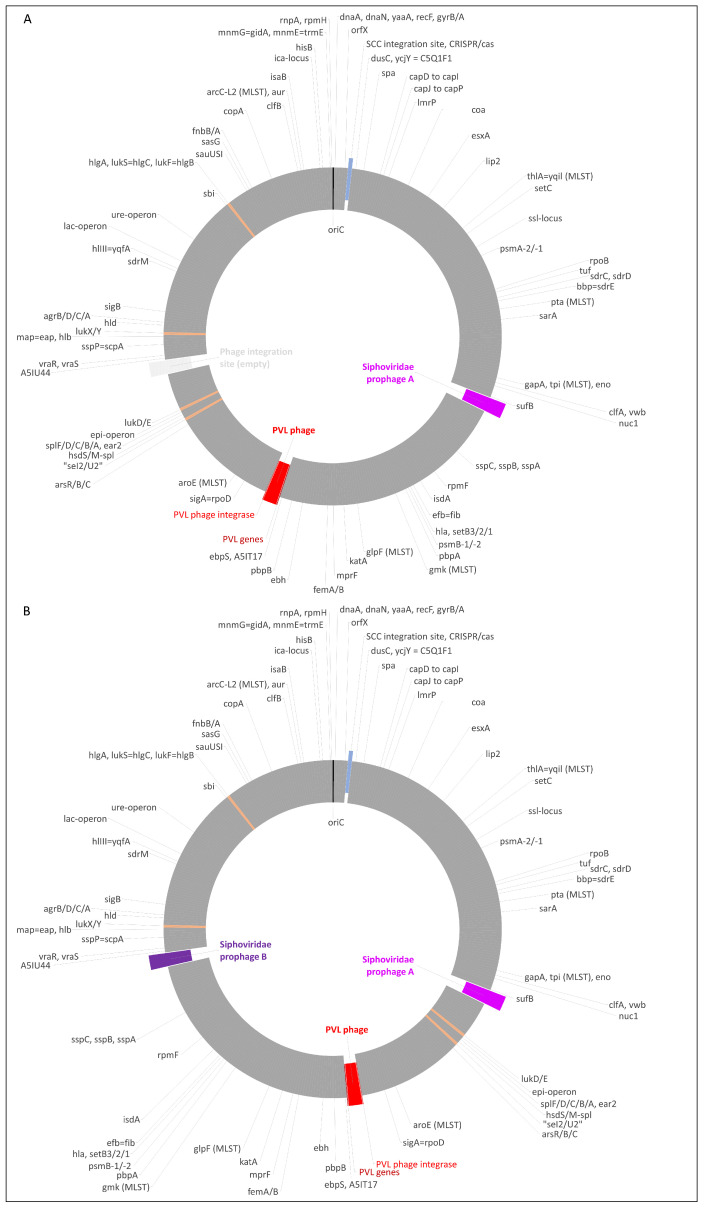
(**A**) Diagram of the genome of *S. argenteus* isolate Dubai-30. The integration site of SCC elements is shown in blue, the PVL prophage in red, and another prophage in purple. The localisations of some other virulence factors are shown in orange, and that of *oriC* in black. Approximately to scale, with one degree corresponding roughly to 8000 nt. This isolate presented with the normal order of core genome markers in *S. aureus* and *S. argenteus.* (**B**) Diagram of the genome of *S. argenteus* isolate Dubai-25. This isolate showed an inversion of the region between the prophages “A” and “B” compared to (**A**).

In the isolate Dubai-25, the entire region between the *sufB*-integrating prophage (“A”) and the A5IU43-integrating prophage (“B”, for details on the prophages, see below) was inverted. This region included also the PVL prophage, hence the inverted order and orientation of its genes, as presented in Table 1. This inverted or flipped region comprised roughly 1,083,000 base pairs, or 38.2% of the strain’s genome (Figure 1A,B). Beyond the issue of its orientation, the regions in question were identical in both strains, differing in only nine single-nucleotide polymorphisms and in 23 positions where single nucleotides were either inserted into one sequence or deleted from the other one.

### 2.3. PVL Prophages

The PVL prophages of the two Dubai isolates were integrated at the same site as PVL prophages in essentially all PVL-positive *S. aureus* strains, which meant dividing the gene A5IT17 (as, for instance, in MW2, BA000033.2, where the two fragments up- and downstream of the prophage carry the locus tags MW1377 and MW1443).

The PVL prophages of the two isolates were found to be essentially identical to each other and to the sequence of phiSa2wa_st78 (GenBank NC_055048; [51]), a PVL (pro-) phage from an Australian CC88/ST78 *S. aureus* isolate, over its entire length of 45,904 base pairs (Supplemental File S2). Differences included a short insertion of 25 bp in both Dubai isolates (into a hypothetical gene with locus tag KMD47_gp04) and a total of 11 SNPs (which were identically present in both Dubai isolates). In addition, there was a missed “A” (in Dubai-30) and an inserted “T” (in both isolates), with these discrepancies affecting poly-A/poly-T-regions, respectively, and this might be attributed to sequencing technology.

**Figure 2:**
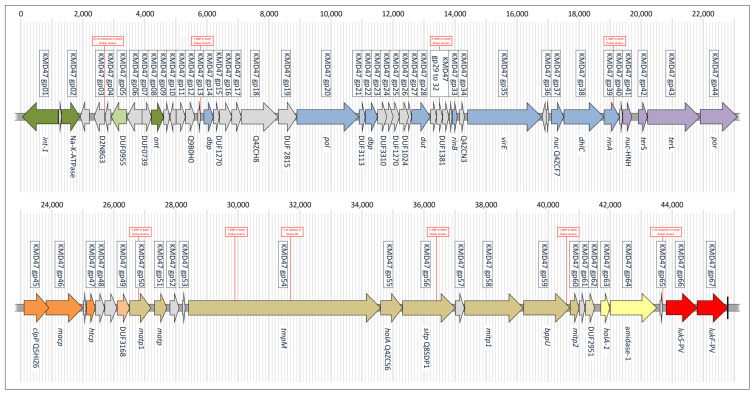
Gene content and orientation of the PVL prophages in the study isolates, and comparison to phiSa2wa_st78 (GenBank NC_055048). For details, see Supplemental File S3.

The genes from the PVL prophages are listed in Figure 2 and in Supplemental File S3, which also display the corresponding locus tags of phiSa2wa_st78 (GenBank NC_055048). It is visible that the two prophage sequences differed in their orientation. This was due to an inversion of a large part of the chromosome of the isolate Dubai-25, as described above.

### 2.4. Other Prophages

The isolate Dubai-25 had, in addition to the PVL prophage, two other prophages. One was integrated into the gene *sufB* (iron–sulphur cluster assembly protein B, cgMLST ID SAUR0895 (SAR_RS04475)), and the other into A5IU43=*yfkAB* (SAUR2054 (SAR_RS10270)). In Dubai-30, only the former position, *sufB*, was occupied. The sequences of these prophages are shown in Supplemental File S2 and their gene content is summarised in Supplemental File S4.

The *sufB*-associated prophages of both isolates were very similar to each other, and they were also related (but not identical) to the prophage that was integrated at the same position in the previously published CC*_arg_*2250 sequence of XNO62, CP023076.1. The A5IU43-associated prophage in Dubai-25 was not identical to either sequence, but showed partial homology. However, it also differed in its orientation. Compared to the *sufB*-associated prophages, it harboured a similar, but not identical, integrase gene. Notably, all these prophages harboured the excisionase gene *xis,* which was not noted in the PVL prophage.

The prophages, despite some similarities (Supplemental Files S2 and S4), differed in their integrase genes. The *sufB*-integrating phages had a 1050 nt integrase gene related to FR821779.1 (873,339…874,388) or CP001781.1 (853,680…854,729), while the A5IU43-integrating phage of Dubai-25 carried a 1047 nt integrase gene related to CP001996.1 (2,017,417…2,018,463) or CP000253.1 (1,965,883…1,966,929).

Both isolates lacked haemolysin-beta-converting phages, and consequently the genes *sak* (staphylokinase), *chp* (chemotaxis-inhibiting protein), *scn* (staphylococcal complement inhibitor), and *sea* or *sep* (enterotoxins A and/or P), which are usually associated with these phages being absent.

### 2.5. Transmission Electron Microscopy of Phages

All phages in the suspensions resulting from Mitomycin C induction experiments on the isolates Dubai-25 and-30 were of the morphotype *Siphoviridae*, characterised by thin, non-contractile tails with a stacked disc appearance and distinct base plates.

**Figure 3:**
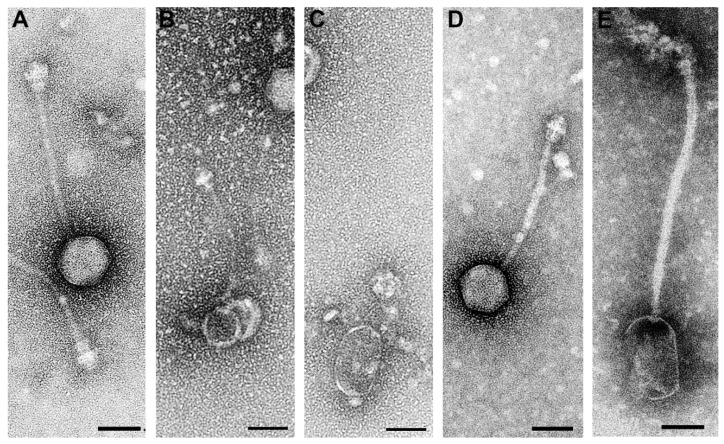
Transmission electron micrograph of representative examples of the large (**A**) and small (**B**) icosahedral phages and of a head of the prolate phages (**C**) from the Dubai-25 preparation, as well as of the icosahedral phages (**D**) and the prolate phage (**E**) from the Dubai-30 preparation. All phages were of the morphotype *Siphoviridae*. Please note that the large icosahedral phages of Dubai-25 (**A**) and Dubai-30 (**D**) have comparable of head and tail measurements and morphology, but distinct shapes of the baseplates. Negative contrast preparation with uranyl acetate (**A**–**D**) and phosphotungstic acid (**E**). Size bars = 50 nm.

During the preparation of Dubai-25, fifteen icosahedral phages with larger heads and 14 icosahedral phages with smaller heads were detected (Table 1, Figure 3A,B). Many of the small icosahedral phages had lost their tails. In addition, there were two prolate phage particles without tails (Figure 3C).

In the Dubai-30 preparation, 20 icosahedral phage particles were measured (Table 1). The size and shape of their heads were comparable with the large icosahedral phages in the preparations from Dubai-25, but less variability was observed in the length of the tails (Table 1, Figure 3D). Baseplates were often obscured by adherent material. While their sizes were comparable, shapes differed between the large icosahedral phages from the Dubai-25 and Dubai-30 preparations (Figure 3A,D). In addition, one prolate phage with a tail which was longer than those of the icosahedral phages was detected in the preparation of Dubai-30 (Figure 3E). Its morphology resembled prolate-headed *Triavirus* specimens (i.e., the genus to which phiSa2wa_st78 was previously assigned [51,54]), allowing the assumption to be made that this was the PVL phage.

### 2.6. Nanopore Sequencing of Phage DNA from Phage Preparations

In addition to TEM studies, it was attempted to sequence phage preparations (Supplemental File S5). It was possible to recover complete sequences of the PVL phages for both strains, although they presented with a number of SNPs and deletions which might have been accidental, i.e., sequencing errors (for an alignment to the prophage sequences, see Supplemental File S5). However, the induced PVL phages from both isolates presented with a ca. 1200 nt deletion encompassing parts of the DNA helicase gene [KMD47_gp38], *rinA*, a gene for a “hypothetical protein” [KMD47_gp40], and an HNH endonuclease gene [KMD47_gp41] (Supplemental File S5).

Nanopore sequencing of a phage preparation from Dubai-25 showed a complete sequence of the A5IU43-integrating phage at a high coverage of 886. The sequence of the *sufB*-integrating phage was not found, which was unexpected given the presence of two distinct populations of icosahedral phages in the TEM studies. Sequencing yielded the sequence of the PVL phage at a low coverage of 11, possibly corresponding to the lower prolate particle count in TEM.

ONT sequencing of a phage preparation from Dubai-30 demonstrated the PVL phage at a coverage of 270. The sequence of the *sufB* phage was also identified, although at a low quality and a coverage of 11, but was followed by a roughly 460,000 nt long fragment of genomic DNA. Its gene content was the same as in the region downstream of the phage in the genome sequence. This might be an assembly error caused by the presence of remnants of chromosomal DNA in the sample.

## 3. Discussion

Temperate phages contribute to the virulence properties of their bacterial hosts, and here, we describe a case in which PVL phages even crossed a species barrier, transmitting PVL genes from *S. aureus* into a *S. argenteus* lineage.

From a purely practical point of view, this observation highlights the need for a correct identification of *S. argenteus* under routine conditions. It also highlights the need for a cheap and rapid, i.e., nonmolecular, test for the detection of the PVL proteins.

PVL-positive *S. argenteus* have previously been observed in Overseas France, Thailand, Myanmar, and the US. Those that have been typed ([45,55] from Myanmar, [19] from Overseas France, and [32] from MO, USA) or that could be typed in retrospect based on published sequences (SAMEA3449074, -3449078, -3449080, -3449083, -3449085, -3449087, -3449096, -3449098, and -3449123 from Thailand) all belonged, like our isolates, to CC*_arg_*2250, while, to the best of our knowledge, no *S. argenteus* from other CCs has been described as positive for PVL. It can be expected that any emerging pathogen, including PVL-positive *S. argenteus,* will sooner or later also be identified in such a cosmopolitan setting as the UAE or in other Arabian Gulf countries, as this region is a global hub for travel, tourism, migration, commerce, and pilgrimage. Furthermore, due to the availability of resources, it is more probable that an emerging pathogen will first be identified in the Arabian Gulf region rather than in the region where it originally emerged. As an extreme example, the MERS virus was first identified in the Arabian Gulf region and not in the Horn of Africa. Similarly, PVL-positive *S. argenteus* might be prevalent in other parts of the world (such as in southeast Asia) but be more readily identified in the region. It might well be expected that strains from other parts of the world might be introduced from elsewhere into this region, but also that returning expatriates or tourists might bring pathogens back into their home countries. The recently observed expansion of a PVL- and *tst1*-positive clone of CC22-MRSA-IVa from the Gulf [56,57] to Nepal [58] and even to China [59] might be such an example. This means that pathogen detection and typing in the Gulf region is even more important than elsewhere.

From a theoretical point of view, it is remarkable that a phage from *S. aureus* might incorporate into an *S. argenteus* genome. The sequence observed was virtually identical to that of phiSa2wa_st78 (GenBank NC_055048), sequenced years earlier from an Australian community-associated MRSA [51]. The differences (13 SNPs and an insertion of 25 nt, on a total of roughly 46,000 nt) are so minuscule that they can be regarded as accidental, possibly even as artefacts. The core genomes of *S. aureus* and *S. argenteus* differ in 11–12% of all nucleotide positions [35], which fully justifies the split into separate species. However, mobile genetic elements, such as SCC*mec* elements [1,2], as well as phages, can cross from one species to the other. This should prompt investigations into more virulence factors that might have spilt over from *S. aureus* into *S. argenteus*, but also for genes that *S. argenteus* might have contributed to the gene pool of *S. aureus.* Virulence factors on phages and other mobile genetic elements that might be observed in both species include not only PVL. There are genes associated with haemolysin-beta-integrating phages *sak, chp,* and *scn* encoding staphylokinase; chemotaxis-inhibiting protein; and staphylococcal complement inhibitors. These are crucial virulence factors in *S. aureus* isolates from humans due to their functional roles in pathogenicity and interactions with the human immune system. Although absent from the isolates described herein, at least the latter three have been observed in *S. argenteus* in various constellations (FR821777.2: *sak, scn*; NZ_CP015758.1: *sak, scn, chp*, SAMEA3449136: *scn* only). Another gene from haemolysin-beta-integrating phages is *sea* encoding enterotoxin A. It has, to the best of our knowledge, not yet been observed in *S. argenteus*, as is also the case for phage-borne exfoliative toxins and the various animal-associated phage-borne leukocidins. The pathogenicity island-associated enterotoxin B gene *seb* occurs occasionally in *S. argenteus,* but appears to be restricted to CC*_arg_*1223. The poorly known enterotoxin-like genes *seli2=sel26* and *selu2=sel27* are also known from other CC*_arg_*2250 and from *S. schweitzeri* (see above), but they also have been found in *S. aureus* CC772, CP010526.1 (1,968,336…1,969,061) and (1,969,088…1,969,840) as well as in CC1956, CP084892 (1,939,378…1,940,121), and (1,940,148…1,940,900). In the two isolates sequenced herein, they were accompanied by phage- or genomic island-associated genes encoding DUF0955 and DUF1433 and by transposase genes, so a cross-species transmission by mobile genetic elements appears to be possible. Again, the improving availability of next-generation sequencing technologies might provide clearer evidence in future.

An interesting question with regard to the incorporation of alien mobile genetic elements is the presence of an *orfX*-associated CRISP/*cas* element in some *S. argenteus*, or more precisely, in essentially all sequences of CC*_arg_*1850 and CC*_arg_*2250 [44]. It is absent from other *S. argenteus* CCs, such as CC*_arg_*1223 [44]. Whether this is related to differences in the ability of different lineages of *S. argenteus* to cope with foreign mobile genetic elements, including PVL phages, still needs to be clarified [44].

In addition to the issue of the PVL prophage, we observed a one-megabase genomic inversion. This is remarkable, as the order of core genomic genes is, first, identical in both *S. aureus* and *S. argenteus* and, second, highly conserved within literally thousands of published genomes. Such a large-scale inversion, to the best of our knowledge, been described only once, in a Russian CC8 MRSA strain [60], where it was linked to a presence of IS256 sequences rather than to prophages. A similar phenomenon was observed during in vitro serial transfer of a *Staphylococcus haemolyticus* strain [61]. In our case, this inversion was not accompanied by a loss of genes or by truncations.

Further studies are needed to explore the intricate interplay between lysogenic phages, virulence genes, and bacterial adaptation in staphylococci populations for better therapeutic strategies.

## 4. Materials and Methods

### 4.1. Characterisation by DNA Microarray Analysis

The microarray-based assays and the related protocols and methods, as well as probe and primer sequences, have been described previously [46,62,63]. In short, enzymatically prepared DNA from overnight cultures was used as template in a site specific linear multiplex primer elongation reaction that incorporated biotin dUTP into the amplicons. These single-stranded, biotin-labelled DNA molecules were stringently hybridised to DNA arrays on which probes for resistance and virulence-associated genes of *S. aureus*, as well as typing markers, were spotted in a pre-defined coordinate grid. Successful hybridisations were visualised by the addition of streptavidin–horseradish–peroxidase, which, in a subsequent step, triggered a localised dye precipitation, resulting in the formation of visible spots at the positions of those probes that bound labelled amplicons. Arrays were photographed and analysed for the presence or absence of specific genes as well as for similarity to known reference profiles using specific threshold values, which facilitated the identification of species, clonal complexes, and strains.

### 4.2. Nanopore Sequencing

The genomes of the two *S. argenteus* isolates (Dubai-25, Dubai-30) were sequenced using the Oxford Nanopore MinION platform to investigate the presence of PVL prophages, whose presence was initially detected using microarray technology.

Bacterial strains were grown on Columbia blood agar (Becton Dickinson, Heidelberg, Germany) overnight at 37 °C. For DNA extraction, the Nucleospin Microbial DNA Kit by Macherey Nagel (MN, Düren, Germany) was utilized.

Each strain was processed by washing one full inoculation loop with 500 μL of 1× PBS (pH 7.4), followed by centrifugation and resuspension in 100 μL of buffer BE. Subsequent procedures adhered to the manufacturer’s protocol with slight adjustments: samples were lysed using a BeatBeater (Biozym, Hessisch Oldendorf, Germany) for 12 min at maximum speed. Proteinase K was deactivated by heating the samples at 70 °C for 5 min. After cooling, 4 μL of RNAse (100 mg/mL; Sigma Aldrich, Steinheim, Germany) was added, and samples were incubated at 37 °C for 5 min before DNA binding onto Nucleospin microbial DNA columns. Finally, DNA was eluted with 70 μL of nuclease-free water (CarlRoth, Karlsruhe, Germany).

For library preparation, the 1D genomic DNA ligation kit (SQK-LSK109, Oxford Nanopore Technologies, Oxford, UK) was employed, following the manufacturer’s instructions for FLO-MIN106 flow cells (FAL13739; Dubai-30) or flongle flow cells (AET365; Dubai-25). Prior to library preparation, size selection was performed using AMPure-beads (Beckman Coulter, Krefeld, Germany) at a 1:1 (*v*/*v*) ratio with the isolated DNA sample. The flow cell for Dubai-30 was loaded with a total of approximately 500 ng DNA (according to Qubit4 Fluorometer; Thermo Fisher Scientific, Waltham, MA, USA). The flongle flow cell for Dubai-25 was loaded with 800 ng total DNA. The sequencing process ran for 72 h using MinKNOW software version 22.12.5 and 22.12.7, starting with a total of around 1200 active pores.

To convert MinION raw reads (FAST5) into quality tagged sequence reads (4000 reads per FASTQ-file), the guppy basecaller (version 6.4.6 + ae70e8f, Oxford Nanopore Technologies) was utilized, with the barcode trimming option enabled (model version: dna_r9.4.1_450bps_sup.cfg and dna_r10.4.1_e8.2_400bps_sup). Flye (version 2.9.1-b1780) was used to assemble the quality tagged sequence reads of each strain into a complete, circular contig. The assemblies underwent two rounds of polishing. First, racon (v1.5.0) was iteratively applied four times with the following parameters: match 8, mismatch 6, gap 8, and window lengths 500. Then, medaka (version 1.7.3) was used on the last racon-polished assembly using the models r941_min_sup_g507 and r10.4.1_e82_400bps_sup_g615. In a final step, Illumina short reads were used for polishing by pilon (version 1.23). The resulting corrected assemblies were used for further analysis.

### 4.3. Phage Induction and Phage DNA Preparation

Phage induction was performed as previously described [64,65,66,67]. Briefly, bacterial cultures were inoculated overnight in 2 × TY medium and cultured at 37 °C until the middle of the exponential growth phase (t = 2 h, OD = 0.68/0.77 for Dubai-25/-30). Mitomycin C (Roche, Basel, Switzerland) was added at a final concentration of 0.5 μg/mL, and cultivation continued at 30 °C until the optical density (OD at 600 nm) began to decrease compared with the previous measurement point (t = 5 h, delta OD = 0.15/1.20 for Dubai-25/-30). The lysate was centrifuged at 4 °C and 3000× *g*, and the supernatant was neutralised with 0.1 N NaOH and filtered using a 0.20 µm cellulose acetate (CA) membrane filter (Sartorius, Göttingen, Germany). To isolate phage DNA (p-DNA), the phage filtrate was centrifuged again for 30 min at 4 °C and 3000× *g*. The resulting supernatant was first treated with 10 µg/mL DNAse I (Sigma Aldrich, Steinheim, Germany) and 10 µg/mL RNAse (QIAGEN, Hilden, Germany) for 1 h at 37 °C. Then, 20 mM EDTA, 50 µg/mL proteinase K, and 0.5% SDS were added sequentially and incubated for another hour at 65 °C and 300 rpm. Phenol–chloroform extraction was then performed as previously described [68]. Phase-lock gel light tubes (Quantabio, Beverly, NJ, USA) were used in each step for better separation of the phases. Finally, the isolated DNA was concentrated in a SpeedVac vacuum concentrator (Eppendorf, Hamburg, Germany) at 1400 rpm and room temperature (20 °C) for 25 min. The final concentration was measured using the Qubit 4 fluorometer (ThermoFisher Scientific, Waltham, MA, USA) according to the manufacturer’s instructions.

### 4.4. Phage Detection by Transmission Electron Microscopy (TEM)

Negative staining was carried out on the phage preparations as previously described [64,65,66]. Briefly, copper grids filmed with formvar, coated with carbon, and hydrophilized by glow discharge were placed on drops of phage preparations for 30 min. After washing with distilled water, one grid of each preparation was contrasted with 1% phosphotungstic acid and one with 1% uranyl acetate for 1 min. Grids were examined using a transmission electron microscope (Tecnai 12, FEI Deutschland GmbH, Dreieich, Germany), and representative micrographs were taken with a digital camera (TEMCAM FX416, TVIPS, Gauting, Germany). Particle size was measured using the EM-Measure software (Version 4.09.53, TVIPS GmbH, Gauting, Germany).

## 5. Conclusions

Temperate bacteriophages contribute to the virulence properties of their bacterial hosts, and here, we describe a case in which a PVL phage even crossed a species barrier, transmitting PVL genes from CC88 *S. aureus* into an *S. argenteus* lineage, CC2250. This observation highlights the need for monitoring emerging staphylococcal strains, especially in cosmopolitan settings such as the UAE; for the correct identification of *S. argenteus* under routine conditions; as well as for a rapid PVL test.

## Figures and Tables

**Table 1:** Phages observed in the preparations from Dubai-25 and Dubai-30.

Isolated From	Shape	Particle Count	Head, in nm ^1^		Tail, in nm ^1^		Baseplate, in nm ^1^	
			Length	Diameter	Length	Diameter	Length	Diameter
Dubai-25	large icosahedral	15	60 ± 3 (54–64)	58 ± 2 (55–63)	162 ± 14 (123–176)	9 ± 1 (7–10)	31 ± 7 (16–37)	25 ± 5 (20–38)
	small icosahedral	14	50 ± 3 (46–54)	49 ± 3 (45–53)	161, 162, 256	7, 8	30, 30, 31	21, 24, 25
	prolate	2	98, 90	53, 54	n.d.	n.d.	n.d.	n.d.
Dubai-30	large icosahedral	20	59 ± 3 (54–63)	58 ± 4 (54–66)	163 ± 5 (152–167)	10 ± 1 (7–12)	28 ± 4 (21–34)	25 ± 3 (20–31)
	prolate	1	98	63	303	10	n.d.	n.d.

^1^ Mean ± standard deviation, followed by range (in brackets); individual measurements are given if particle numbers were less than 5; n.d., not detected.

## Data Availability

All relevant data are provided as Supplementary Materials. The sequences of the genomes have been submitted to GenBank (BioProject ID: PRJNA1092990; BioSample accessions: SAMN40643810, SAMN40643811; GenBank accession numbers pending).

## Supplementary Materials

The following supporting information can be downloaded at: https://www.mdpi.com/article/10.3390/antibiotics13050401/s1; Supplemental File S1: Genome Sequences of the study strains (fasta); Supplemental File S2: Phages from Dubai 25, Dubai_30, and phiSa2wa_st78, NC_055048 (fasta); Supplemental File S3: Gene content of PVL prophages in isolates Dubai-25 and Dubai-30 (pdf); Supplemental File S4: Gene content of the other prophages in isolates Dubai-25 and Dubai-30 and comparison to a *sufB*-integrating prophage from another CC_arg_2250 strain, XNO62 CP023076.1 (pdf); Supplemental File S5: ONT phage sequences obtained directly from Mitomycin C-induced phage preparations (fasta).
