# Supplementary material for: Characterisation of PVL-Positive Staphylococcus argenteus from the United Arab Emirates
Source: Antibiotics (Basel). 2024 Apr 27;13(5):401. doi: 10.3390/antibiotics13050401 (PMC11117363; doi:10.3390/antibiotics13050401)
Supplement: Supplementary file 1 [file antibiotics-13-00401-s001.zip › Supplemental File 3_Gene content of PVL prophages in isolates Dubai-25 and Dubai-30.pdf]

Supplemental File 3: Gene content of PVL phages in isolates Dubai-25 and Dubai-30 (FRWRD=Forward, RVRCO=Reverse complement).

Gene ID	Gene Product/Description	Locus tag in NC_055048	Start in Dubai-25	End in Dubai-25	Orientation in Dubai-25	Start in Dubai-30	End in Dubai-30	Orientation in Dubai-30
int-1	integrase	KMD47_gp01	1299793	1300998	RVRCO	1586371	1587576	FRWRD
N/A	hypothetical protein associated with phage integrase	Present but not annotated	1300997	1301127	FRWRD	1586242	1586372	RVRCO
phi-dut	Na/K ATPase	KMD47_gp02	1301124	1301738	FRWRD	1585631	1586245	RVRCO
N/A	hypothetical protein, phi-3A-ORF130	Present but not annotated	1301735	1301860	RVRCO	1585509	1585634	FRWRD
phi-D2N8G3	hypothetical protein	KMD47_gp03 *plus* KMD47_gp04	1302111	1302695	RVRCO	1584674	1585258	FRWRD
DUF0955	metallo-protease	KMD47_gp05	1302712	1303170	RVRCO	1584199	1584657	FRWRD
N/A	hypothetical protein	KMD47_gp06	1303192	1303668	RVRCO	1583701	1584177	FRWRD
DUF0739	DUF739 family protein	KMD47_gp07	1303710	1303958	FRWRD	1583411	1583659	RVRCO
ant	anti-repressor Ant	KMD47_gp08	1303976	1304419	FRWRD	1582950	1583393	RVRCO
N/A	hypothetical protein	KMD47_gp09	1304434	1304577	FRWRD	1582792	1582935	RVRCO
N/A	hypothetical protein	KMD47_gp10	1304567	1304785	RVRCO	1582584	1582802	FRWRD
N/A	transcriptional regulator	KMD47_gp11	1304820	1305077	FRWRD	1582292	1582549	RVRCO
Q9B0H0	hypothetical protein	KMD47_gp12	1305046	1305411	RVRCO	1581958	1582323	FRWRD
N/A	hypothetical protein	KMD47_gp13	1305466	1305603	FRWRD	1581766	1581903	RVRCO
dbp	DNA binding protein	KMD47_gp14	1305697	1305969	FRWRD	1581400	1581672	RVRCO
DUF1270	DUF1270 domain-containing protein	KMD47_gp15	1305981	1306142	FRWRD	1581227	1581388	RVRCO
N/A	transcriptional regulator	KMD47_gp16	1306221	1306544	FRWRD	1580825	1581148	RVRCO
N/A	hypothetical protein	KMD47_gp17	1306559	1306921	FRWRD	1580448	1580810	RVRCO
Q4ZCH8=DUF2800	DUF2800 domain-containing protein, nuclease superfamily protein from Siphoviridae	KMD47_gp18	1306918	1308084	FRWRD	1579285	1580451	RVRCO
DUF2815	Gp2.5-like ssDNA binding protein and ssDNA annealing protein	KMD47_gp19	1308065	1308667	FRWRD	1578702	1579304	RVRCO
pol_phi12/phi2638A	DNA polymerase	KMD47_gp20	1308726	1310687	FRWRD	1576682	1578643	RVRCO
DUF3113	DUF3113 family protein	KMD47_gp21	1310700	1310885	FRWRD	1576484	1576669	RVRCO
dbp	putative DNA-binding/PVL-phage ORF50-like protein	KMD47_gp22	1310882	1311286	FRWRD	1576083	1576487	RVRCO
DUF3310	nucleotide kinase	KMD47_gp23	1311286	1311543	FRWRD	1575826	1576083	RVRCO

Gene ID	Gene Product/Description	Locus tag in NC_055048	Start in Dubai-25	End in Dubai-25	Orientation in Dubai-25	Start in Dubai-30	End in Dubai-30	Orientation in Dubai-30
N/A	hypothetical protein, MW1421	KMD47_gp24	1311531	1311746	FRWRD	1575623	1575838	RVRCO
DUF1270	virulence associated protein	KMD47_gp25	1311740	1312003	FRWRD	1575366	1575629	RVRCO
DUF1024	DUF1024 family protein	KMD47_gp26	1312009	1312272	FRWRD	1575097	1575360	RVRCO
N/A	hypothetical protein	KMD47_gp27	1312241	1312429	FRWRD	1574940	1575128	RVRCO
dut	dUTP pyrophosphatase, replication module of Siphoviridae	KMD47_gp28	1312422	1312949	FRWRD	1574420	1574947	RVRCO
N/A	hypothetical protein	KMD47_gp29	1312986	1313159	FRWRD	1574210	1574383	RVRCO
DUF1381	transcriptional regulator	KMD47_gp30	1313176	1313382	FRWRD	1576484	1576669	RVRCO
N/A	hypothetical protein	KMD47_gp31	1313370	1313573	FRWRD	1573796	1573999	RVRCO
N/A	hypothetical protein	KMD47_gp32	1313570	1313722	FRWRD	1573647	1573799	RVRCO
rinB	RinB-like transcriptional activator	KMD47_gp33	1313688	1313867	FRWRD	1573502	1573681	RVRCO
Q4ZCN3=DUF1514	hypothetical protein	KMD47_gp34	1313902	1314135	FRWRD	1573234	1573467	RVRCO
virE	DNA helicase	KMD47_gp35	1314187	1316634	FRWRD	1570735	1573182	RVRCO
N/A	hypothetical protein, ST42eORF147	Present but not annotated	1316658	1316780	RVRCO	1570589	1570711	FRWRD
N/A	hypothetical protein	KMD47_gp36	1316697	1316801	FRWRD	1570568	1570672	RVRCO
N/A	hypothetical protein, UG86_01560	Present but not annotated	1316743	1316832	RVRCO	1570537	1570626	FRWRD
nuc_Q4ZCF7	endonuclease	KMD47_gp37	1316915	1317265	FRWRD	1570104	1570454	RVRCO
dhlC-1	DNA helicase	KMD47_gp38	1317246	1318613	FRWRD	1568756	1570123	RVRCO
rinA=Q4ZCF5	transcriptional regulator	KMD47_gp39	1318626	1319063	FRWRD	1568306	1568743	RVRCO
N/A	hypothetical protein	KMD47_gp40	1319108	1319233	FRWRD	1568136	1568261	RVRCO
nuc-HNH	HNH endonuclease	KMD47_gp41	1319220	1319534	FRWRD	1567835	1568149	RVRCO
terS	terminase small subunit	KMD47_gp42	1319643	1319966	FRWRD	1567403	1567726	RVRCO
terL	terminase large subunit	KMD47_gp43	1319947	1321647	FRWRD	1565722	1567422	RVRCO
por	portal protein	KMD47_gp44	1321652	1322890	FRWRD	1564479	1565717	RVRCO
clpP=Q5HIZ6=pro	Clp-protease/"head maturation protein", packaging module of Siphoviridae	KMD47_gp45	1322862	1323647	FRWRD	1563722	1564507	RVRCO
macp=mcp	major capsid protein	KMD47_gp46	1323614	1324822	FRWRD	1562547	1563755	RVRCO

Gene ID	Gene Product/Description	Locus tag in NC_055048	Start in Dubai-25	End in Dubai-25	Orientation in Dubai-25	Start in Dubai-30	End in Dubai-30	Orientation in Dubai-30
N/A	hypothetical protein, ST42eORF172	Present but not annotated	1324770	1324880	FRWRD	1562489	1562599	RVRCO
htcp	head-tail adaptor Ad1	KMD47_gp47	1324891	1325169	FRWRD	1562200	1562478	RVRCO
N/A	hypothetical protein	KMD47_gp48	1325181	1325513	FRWRD	1561856	1562188	RVRCO
N/A	hypothetical protein, SAR1513, SACOL0373	Present but not annotated	1325540	1325911	FRWRD	1561458	1561829	RVRCO
DUF3168	DUF3168 domain-containing protein	KMD47_gp49	1325912	1326307	FRWRD	1561062	1561457	RVRCO
matp1 =mtp	tail protein	KMD47_gp50	1326342	1326983	FRWRD	1560386	1561027	RVRCO
matp	major tail protein	KMD47_gp51	1327075	1327530	FRWRD	1559839	1560294	RVRCO
N/A	putative protein, tail module of Siphoviridae	KMD47_gp52	1327588	1327929	FRWRD	1559440	1559781	RVRCO
N/A	hypothetical protein	KMD47_gp53	1327980	1328138	FRWRD	1559231	1559389	RVRCO
tmpM	tail length tape measure protein	KMD47_gp54	1328152	1334352	FRWRD	1553018	1559217	RVRCO
holA=Q4ZCS6	putative bacteriophagal tail protein/holin	KMD47_gp55	1334352	1335176	FRWRD	1552194	1553018	RVRCO
sitp=Q8SDP1	tail protein with endopeptidase domain	KMD47_gp56	1335185	1336768	FRWRD	1550602	1552185	RVRCO
N/A	hypothetical protein	KMD47_gp57	1336768	1337058	FRWRD	1550312	1550602	RVRCO
mitp1	minor tail protein	KMD47_gp58	1337074	1338984	FRWRD	1548386	1550296	RVRCO
bppU=DUF2479	BppU family baseplate upper protein	KMD47_gp59	1338984	1340450	FRWRD	1546920	1548236	RVRCO
mitp2=DUF2977	tail fiber protein	KMD47_gp60	1340450	1340839	FRWRD	1546531	1546920	RVRCO
N/A	tail fiber protein	KMD47_gp61	1340832	1340996	FRWRD	1546374	1546538	RVRCO
DUF2951=Q9MBN6	DUF2951 domain-containing protein	KMD47_gp62	1341021	1341341	FRWRD	1546029	1546349	RVRCO
holA-1	holin, lysis module of Siphoviridae	KMD47_gp63	1341432	1341779	FRWRD	1545591	1545938	RVRCO
ami	N-acetylmuramoyl-L-alanine amidase, lysis module of Siphoviridae	KMD47_gp64	1341790	1343244	FRWRD	1544126	1545580	RVRCO
O80065	hypothetical protein accompanying PVL genes	KMD47_gp65	1343394	1343526	FRWRD	1543844	1543976	RVRCO
lukS-PV	Panton-Valentine leukocidin, S component	KMD47_gp66	1343625	1344572	FRWRD	1542798	1543745	RVRCO
lukF-PV	Panton-Valentine leukocidin, F component	KMD47_gp67	1344574	1345551	FRWRD	1541819	1542796	RVRCO
