# Supplementary material for: Characterisation of PVL-Positive Staphylococcus argenteus from the United Arab Emirates
Source: Antibiotics (Basel). 2024 Apr 27;13(5):401. doi: 10.3390/antibiotics13050401 (PMC11117363; doi:10.3390/antibiotics13050401)
Supplement: Supplementary file 1 [file antibiotics-13-00401-s001.zip › Supplemental File 4_Gene content of the other prophages in isolates Dubai-25 and Dubai-30.pdf]

Supplemental File 4: Gene content of the other prophages in isolates Dubai-25 and Dubai-30 and comparison to *a sufB*-integrating prophage from another CC_{arg}2250 strain, XNO62 CP023076.1

	**Dubai-25 [sufB-integr prophage]**		**Dubai-30 [sufB-integr prophage]**		**Dubai-25 [A5IU43-integr prophage]**		**CP023076.1:847404-893404 [sufB-integr prophage]**	
	Start pos.	End pos.	Start pos.	End pos.	Start pos.	End pos.	Start pos.	End pos.
phi-int6 (A5IU43)					2025668	2026714		
phi-int6 (sufB)	858470	859519	858495	859544			847404	848454
phi-xis (180 nt)	859632	859811	859657	859836	2025376	2025555		
phi-xis (202 nt)							848565	848766
hypothet. phage protein							848702	849608
hypothet. phage protein RU53_629					2005295	2005597		
phi-Q932C1	859791	860723	859816	860748	2024464	2025396		
phi-A6U3A7					2024157	2024324		
phi-A6U3A7_SPV-80A_gp04							849643	849829
hypothet. phage protein	860755	861480	860780	861505				
-rRNA_binding protein	861508	862182	861533	862207				
phi-TransReg	862199	862531	862224	862556				
phi-xrep_SACOL0321					2023368	2024087		
phi-xrep_Sipho-0162							850024	850185
hypothet. phage protein							850490	850643
hypothet. phage protein							850921	851137
phi-xrep (186nt)							851898	852084
phi-xrep (195 nt)	862794	862988	862819	863013				
phi-cap-Q8NWW7					2023008	2023226		
phi-ant_FIV54_08865	862988	863752	863013	863777				
phi-ant_							852085	852835
phi-ant_SAB1755					2022216	2022992		
hypothet. phage protein phi29-ORF091							852850	853066

	**Dubai-25 [sufB-integr prophage]**		**Dubai-30 [sufB-integr prophage]**		**Dubai-25 [A5IU43-integr prophage]**		**CP023076.1:847404-893404 [sufB-integr prophage]**	
	Start pos.	End pos.	Start pos.	End pos.	Start pos.	End pos.	Start pos.	End pos.
SARLGA251_07830							853021	853267
phi-Q931J5	863769	863963	863794	863988	2022005	2022199		
phi-treG_CA347_1950	864786	865007	864811	865032			853337	853559
phi-treG_ST42eORF072					2021388	2021606		
phi-DUF1270_SAS063	865000	865161	865025	865186	2021234	2021395		
hypothet. phage protein ROSA-ORF193	865020	865130	865045	865155			853571	853682
phi-DUF1108_	865253	865513	865278	865538			853804	854065
phi-AAA-ATPase							854558	855332
NW943_03595							856675	857335
phi-Q4ZAK4_rep							857380	858151
phi-DUF2483_SACOL0337	865523	865744	865548	865769	2020652	2020873		
phi-ssaP_DUF1071	865737	866360	865762	866385	2020036	2020659		
phi-ssbP_C9J86_00295	866360	866788	866385	866813	2019608	2020036		
phi-DUF0968_SACOL0340					2018899	2019594		
phi-DUF0968_Sipho-0780to83_SAOV_1954c	867470	868249	867495	868274				
phi-Q4ZAY9_SaO11_01748	868257	869024	868282	869049				
phi-Q4ZAY9_SACOL0341					2018109	2018927		
phi-dbp_SACOL0342					2017753	2018109		
phi-dnaC=istB1_SaO11_01747	869034	869807	869059	869832			858160	858934
phi-sri	869801	869959	869826	869984			858927	859086
phi-DUF3269_ SACOL0345	869972	870193	869997	870218			859098	859320
phi-DUF1064_SAOV_1947c	870203	870607	870228	870632			859329	859734
phi-dhlC-2a_SACOL0343					2016515	2017756		
hypothet. phage protein SACOL0344					2016303	2016518		
phi-DUF3113_SACOL0348	870612	870797	870637	870822				

	Dubai-25 [sufB-integr prophage]		Dubai-30 [sufB-integr prophage]		Dubai-25 [A5IU43-integr prophage]		CP023076.1:847404-893404 [sufB-integr prophage]	
	Start pos.	End pos.	Start pos.	End pos.	Start pos.	End pos.	Start pos.	End pos.
phi-DUF3113_MW1424					2016079	2016300		
phi-DUF1064_SACOL0347					2015664	2016068		
phi-dbp_MW1917	870798	871160	870823	871185	2015108	2015470	859927	860287
phi-DUF1270_ST0131_02112	871245	871409	871270	871434	2014859	2015023	860368	860533
phi_gnaT_SAA6159_01912	871457	871789	871482	871814				
phi-DUF1024_Sipho	871782	872024	871807	872049	2014356	2014844	860547	860796
hypothet. phage protein MW1418							860788	860962
hypothet. phage protein MW1417							860962	861244
hypothet. phage protein MW1416							861244	861406
phi-dut_SaO11_00288							861555	861948
phi-dut_SACOL0357	872259	872768	872284	872793				
phi-dut_SAOV_1092					2013821	2014363		
phi-DUF1381_KMD47_gp30							861984	862191
hypothet. phage protein MW1412					2013421	2013621		
hypothet. phage protein SAB1731c	872805	873050	872830	873075				
phi-pol_NW963_09520	873308	873544	873333	873569				
phi-DUF1523_SAKOR_01952	873537	873923	873562	873948				
phi-DUF1381_CU118_04855					2013596	2013784		
phi-rinB_SATW20_03570	873920	874093	873945	874118	2013245	2013418		
phi-rinM	874097	874240	874122	874265	2013098	2013241	862364	862508
phi-rinA	874255	874674	874280	874699	2012652	2013074	862522	862942
phi-terS_MS7_0616	874861	875301	874886	875326	2012025	2012465	863128	863569
phi-terL_MS7_0617	875288	876565	875313	876590	2010761	2012038		
phi-terL							863555	864833
phi-port_A4ZFA8	876576	878111	876601	878136	2009215	2010750	864843	866382
phi-micp/hmp_SAB1724c	878232	879113	878257	879138	2008213	2009094	866502	867384

	Dubai-25 [sufB-integr prophage]		Dubai-30 [sufB-integr prophage]		Dubai-25 [A5IU43-integr prophage]		CP023076.1:847404-893404 [sufB-integr prophage]	
	Start pos.	End pos.	Start pos.	End pos.	Start pos.	End pos.	Start pos.	End pos.
hypothet. phage protein NCTC5663_00903	879186	879356	879211	879381			867456	867627
phi-DUF4355_scaf_SAB1723c	879491	880105	879516	880130			867761	868376
phi-DUF4355_scaf_phi-69-ORF022					2007232	2007852		
phi-macp_phi-69-ORF011	880119	881093	880144	881118	2006244	2007218	868389	869364
hypothet. phage protein SAB1721c	881115	881402	881140	881427	2005935	2006222	869385	869673
phi-htcp_SAB1720c			881436	881768			869681	870014
phi-htcp	881411	881743			2005594	2005926		
hypothet. phage protein SAB1719c	881740	882042	881765	882067			870010	870313
phi-tail	882042	882389	882067	882414	2004948	2005295	870312	870660
phi-matp_MS7_0627			882828	883409			871073	871655
hypothet. phage protein			882967	883071			871212	871317
hypothet. phage protein SAB1717c	882401	882784			2004553	2004936	870671	871055
phi-DUF3647_tmchap_KI244_01915	883447	883812			2003525	2003890		
hypothet. phage protein SAB1714c	883842	884186	883866	884210	2003151	2003495	872111	872456
phi-tmpM5_ML435_08605	884203	887667	884227	887691	1999667	2003134	872472	875937
phi-stp	887680	888627	887704	888651	1998707	1999654	875949	876897
hypothet. phage protein			890419	890583			878664	878829
phi-mitp1			890576	892486			878821	880732
phi-Q2FX67=pep_SAB1711c			888660	890561				
phi-Q2FX67=pep_SAOUHSC_02030	888636	890537			1996797	1998698	876905	878807
hypothet. phage protein NCTC5663_00917	890552	892462						
hypothet. phage protein SAOV_1958c					1994872	1996782	854079	854559
phi-bppU/mitp2	892462	894285	892486	894309	1993049	1994872	880731	882555
phi-mitp2=DUF2977_CA347_1906	894285	894662	894309	894686	1992672	1993049	882554	882932

	Dubai-25 [sufB-integr prophage]		Dubai-30 [sufB-integr prophage]		Dubai-25 [A5IU43-integr prophage]		CP023076.1:847404-893404 [sufB-integr prophage]	
	Start pos.	End pos.	Start pos.	End pos.	Start pos.	End pos.	Start pos.	End pos.
phi-xldX_MS7_0636	894666	894839	894690	894863	1992495	1992668	882935	883109
phi-Q9MBN6=DUF2951	894880	895179	894904	895203	1992155	1992454	883149	883449
phi-hydr	895316	897214	895340	897238	1990120	1992018	883585	885484
phi-bppU/tailfiber-2	897227	898399	897251	898423	1988935	1990107	885496	886670
hypothet. phage protein phi-53-ORF098	897301	897429	897325	897453	1989905	1990033	885570	885699
hypothet. phage protein	898405	898800	898429	898824	1988534	1988929	886675	887071
phi-holA-1_SACOL0388							887126	887429
phi-holA-3_SAOV_1121	898856	899293	898880	899317	1988041	1988478		
phi-amidase-3_SAPIG1492	899274	900719	899298	900743	1986615	1988060		
phi-amidase							887439	888894
hypothet. phage protein phi-92-ORF113	900355	900510	900379	900534	1986824	1986979		
hypothet. phage protein LB315_05740	900963	901115	900987	901139	1986219	1986371		
hypothet. phage protein NCTC6131_00991	900987	901115	901011	901139	1986219	1986347		
hypothet. phage protein SACOL0910	901186	901296	901210	901320	1986038	1986148		
phi-Q2YXQ4	901283	901483	901307	901507	1985851	1986051		
EBI08_07095							888726	888885
hypothet. phage protein							888559	888691
hypothet. phage protein ST42eORF161							889069	889171
A7971_09590							889851	890409
Q2YWM5							891024	891339
